# Prevalence and determinants of maternal healthcare utilisation among young women in sub-Saharan Africa: cross-sectional analyses of demographic and health survey data

**DOI:** 10.1186/s12889-022-13037-8

**Published:** 2022-04-05

**Authors:** Luchuo Engelbert Bain, Richard Gyan Aboagye, Robert Kokou Dowou, Eugene Justine Kongnyuy, Peter Memiah, Hubert Amu

**Affiliations:** 1 College of Social Science, Lincoln International Institute for Rural Health (LIIRH), University of Lincoln, Lincoln, UK; 2 Department of Family and Community Health, School of Public Health, University of Health and Allied Sciences, Hohoe, Ghana; 3 Department of Epidemiology and Biostatistics, School of Public Health, University of Health and Allied Sciences, Hohoe, Ghana; 4 United Nation’s Population Fund, UNFPA, Bamako, Mali; 5 Division of Epidemiology and Prevention: Institute of Human Virology, University of Maryland School of Medicine, Baltimore Maryland, USA; 6 Department of Population and Behavioural Sciences, School of Public Health, University of Health and Allied Sciences, Hohoe, Ghana

**Keywords:** Sub-Saharan Africa, Antenatal Care, Skilled Birth Attendance, Postnatal Care, Maternal Healthcare Utilisation, Sustainable Development Goals

## Abstract

**Background:**

Maternal health constitutes high priority agenda for governments across the world. Despite efforts by various governments in sub-Saharan Africa (SSA), the sub-region still records very high maternal mortality cases. Meanwhile, adequate utilization of maternal healthcare (antenatal care [ANC], skilled birth attendance [SBA], and Postnatal care [PNC]) plays a vital role in achieving improved maternal health outcomes. We examined the prevalence and determinants of maternal healthcare utilization among young women in 28 sub-Saharan African countries using data from demographic and health surveys.

**Methods:**

This was a cross-sectional study of 43,786 young women aged 15–24 years from the most recent demographic and health surveys of 28 sub-Saharan African countries. We adopted a multilevel logistic regression analysis in examining the determinats of ANC, SBA, and PNC respectively. The results are presented as adjusted Odds Ratios (aOR) for the logistic regression analysis. Statistical significance was set at *p* < 0.05.

**Results:**

The prevalence of maternal healthcare utilisation among young women in SSA was 55.2%, 78.8%, and 40% for ANC, SBA, and PNC respectively with inter-country variations. The probability of utilising maternal healthcare increased with wealth status. Young women who were in the richest wealth quintile were, for instance, 2.03, 5.80, and 1.24 times respectively more likely to utilise ANC (95% CI = 1.80–2.29), SBA (95% CI = 4.67–7.20), and PNC (95% CI = 1.08–1.43) than young women in the poorest wealth quintile. Young women who indicated having a barrier to healthcare utilisation were, however, less likely to utilise maternal healthcare (ANC: aOR = 0.83, 95% CI = 0.78–0.88; SBA: aOR = 0.82, 95% CI = 0.75–0.88; PNC: aOR = 0.88, 95% CI = 0.83–0.94).

**Conclusion:**

While SBA utilisation was high, we found ANC and PNC utilisation to be quite low among young women in SSA with inter-country variations. To accelerate progress towards the attainment of the Sustainable Development Goal (SDG) targets on reducing maternal mortality and achieving universal health coverage, our study recommends the adoption of interventions which have proven effective in some countries, by countries which recorded low maternal healthcare utilisation. The interventions include the implementation of free delivery services, training and integration of TBAs into orthodox maternal healthcare, improved accessibility of facilities, and consistent public health education. These interventions could particularly focus on young women in the lowest wealth quintile, those who experience barriers to maternal healthcare utilisation, uneducated women, and young women from rural areas.

**Supplementary Information:**

The online version contains supplementary material available at 10.1186/s12889-022-13037-8.

## Background

Maternal health constitutes high priority agenda for governments across the world. It is an important component of the global Sustainable Development Goals (SDGs) set in 2015 by the United Nations [1–3] SDG targets 3.1 and 3.8, for instance, seek to reduce the maternal mortality ratio to less than 70 maternal deaths per 100,000 live births, and achieve universal health coverage respectively. Despite efforts by various governments in Sub-Saharan Africa (SSA), the sub-region still records very high maternal mortality cases. A World Health Organisation (WHO) report [2], for instance, reported that more than 800 maternal mortality cases occur each day in SSA due to pregnancy-related and childbirth-related complications.

Adequate utilisation of maternal healthcare which constitutes Antenatal care (ANC), Skilled Birth Attendance (SBA), and postnatal care (PNC), which are proven services that play a vital role in achieving improved maternal health outcomes [4], has been recognised as the panacea in mitigating the menace of maternal mortality in SSA [5–8]. For instance, timely and appropriate antenatal utilisation alone can reduce maternal mortality by 20% [9]. Similarly, PNC utilisation within 24 h after birth as recommended by WHO is crucial for averting maternal deaths [10]. PNC stage mornally commence immediately after childbirth until 42 days after birth [11]. According to recommendation by WHO women should receive at least three postnatal care visits in addition to the first visit which is expected to take place within 24 h after birth [12].

Despite the importance of maternal healthcare utilisation in promoting maternal health, coverage in SSA is still quite low. For instance, irrespective of its cruicial role in the reducing maternal mortality, large number of women continue to give birth without professional assistance in some SSA countries, especially during their subsequent deliveries. In Ethiopia for instance, approximately 70.8% of women gave birth without any assistance of SBA at home during the last child birth [13].

Studies conducted at individual country levels have revealed that socio-economic status, availability and accessibility to health facility, knowledge on pregnancy emergencies, and educational level of mothers influence the maternal service utilisation in SSA [14–16].

Several interventions have been implemented by SSA countries to improve the maternal health service utilisation [17–20]. Ghana for example introduced one most important health financing reform in the history of the country, free maternal healthcare policy (FMHCP) in 2008 as part of the Ghana National Health Insurance Scheme (NHIS) to removing financial barriers and resultant inequalities in maternal healthcare utilisation among pregnant and nursing mothers [17, 21, 22]. This policy has increased access and utilisation among mothers [14]. According to Novignon, Ofori, Tabiri, and Pulok [17], with the implementation of the policy, ANC has increased from 70.6% in 2003 to 86.5% in 2014. Similarly, skilled birth attendance increased from 43.9% in 2003 to 72.8% in 2014 [17].

The majority of studies carried out in SSA on maternal healthcare utilisation have not combined all the three components (ANC, PNC and skilled delivery). The combination of the three services in this study provides an opportunity to make direct comparison among the maternal services and also determine how these services influences each other when combined. The studies have also focused on individual country levels. To bridge this existing gap, we analysed ANC, SBA, and PNC using the nationality representative demographic and health survey (DHS) data on young women using data from 28 countries in SSA. This study focused on young women aged 15–24 because they have the highest risks of developing obstetric complications such as sepsis, postpartum haemorrhage, pregnancy-induced hypertension, and associated mortalities [23, 24]. Understanding the maternal healthcare utilisation patterns in this priority age bracket is relevant in providing targeted healthcare interventions. The study also adopted multivariable logistic regression to achieve a robust analysis of the various determinants (individual and contextual) influencing maternal healthcare utilisation among young women in SSA. The findings could inform the formulation and implementation of interventions focused on maternal healthcare utilisation in the sub-region.

## Materials and methods

### Data source and study design

This study involved a cross-sectional analysis of data from Demographic and Health Surveys (DHS) of twenty-eight (28) countries in SSA. Data for the study were pooled from the most recent surveys in those countries, specifically the women’s file. DHS is a nationally representative survey usually conducted every 5 years in over 85 low- and middle-income countries [25]. The DHS uses a structured questionnaire to collect data from the respondents on health indicators such as maternal and child health [25]. The survey employed a two-stage sampling method to collect data from the respondents. A detailed explanation of the sampling process and data collection methodology has been published elsewhere [26]. A total of 43,786 young women aged 15–24 years with complete cases of variables of interest were included in the final analysis. The sample size per country can be found in Appendix 1. We relied on the Strengthening the Reporting of Observational Studies in Epidemiology (STROBE) statement in writing the manuscript [27] (See Appendix 2).

### Study variables

#### Outcome variable

The outcome variable in the present study was maternal healthcare service utilisation. This variable has three main components consisting of ANC, SBA, and PNC. Regarding the utilisation of ANC, the women were asked about the number of antenatal visits they made during their recent pregnancy. The responses were recoded as 0–3 = 0 “No" and 4 and above = 1 “Yes”. With SBA, the women were asked “Who assisted [NAME] during delivery?”. The response to this question was categorised into “Traditional Birth Attendant/Others” = 0 and “SBA/Health professionals” = 1. Also, PNC attendance was derived from the question, “Did [NAME] go for PNC checks within 2 months?”. The response options were “Yes”, “No” and “Don’t know”. This was recoded into “No” = 0 and “Yes” = 1. The categorizations and recodings used in the present study were informed by literature [28–30].

#### Explanatory variables

A total of 16 explanatory variables were included in the study. These variables were selected because of their association with the outcome variables from previous studies [28, 29, 31–33] as wells as their availability in the DHS dataset. The variables were further grouped in to individual level and household/community (contextual) level. The individual level variables included age of the women and the partner or husband (years), education level of the respondent and the husband, marital status, religion, current working status, parity, exposure to mass media, health insurance ownership, person who usually decides on respondents healthcare, person who usually decides on large household purchases, and person who usually decides on visit to family or relatives. The survey years of the datasets used were controlled for as individual level variables. The household/community level variables consisted of wealth index, sex of household head, place of residence, and geographic subregions. For the individual level variables, maternal age was recoded as “15–19” and “20–24” years respectively. Marital status was recoded as “married” and “cohabiting”. Both the maternal and partner educational levels maintained the existing coding in the DHS dataset which was “no education”, “primary”, “secondary”, and “higher”. Religion was recoded as “Christianity”, “Islamic”, “African Traditional”, “no religion, and “others”. Partner age was recoded as “15–24”, “25–34”, “35–44”, and “45 and above”. Maternal working status was recoded as “not working” and “working”. Parity was recoded as “1”, “2”, “3”, and “4 or more”. We maintained the already existing coding for national health insurance (“No” and “yes’) as found in the DHS dataset. Exposure to media was created from three (3) variables (frequency of watching television, frequency of reading newspaper/magazine, and frequency of listening to the radio). All three variables had the same response options (not at all, less than once a week, at least once a week, and almost every day). The women who responded not at all were categorised as “Not exposed to mass media [No]” whilst those whose responses were less than once a week, at least once a week, and almost everyday were grouped as “exposed to mass media [Yes]”. An index variable called mass media exposure was created using the recoded responses from the three variables. Any woman with at least exposure from one of the variables was set to have exposure to mass media. The barrier to healthcare was created from three questions which consisted of difficulty in obtaining money (money), distance to health facility (distance), getting permission for treatment (permission). Any woman with at least “Yes” in any of the three was categorised as having a barrier to healthcare. Person who usually decides on respondents healthcare, person who usually decides on large household purchases, and person who usually decides on visit to family or relatives were coded as (“respondent alone”, “respondent and husband/partner”, “partner alone”, and “someone else or other”) respectively. For the household/community level variables, wealth index (“poorest”, “poorer”, “middle”, “richer”, and “richest”), sex of household head (“male” and “female”), and place of residence (“urban” and “rural”) as coded in the DHS dataset were maintained and used in the final analysis. Geographical subregion was coded as (“Southern”, “Central”, “Eastern”, and “Western”).

#### Statistical analyses

**Fig. 1:**
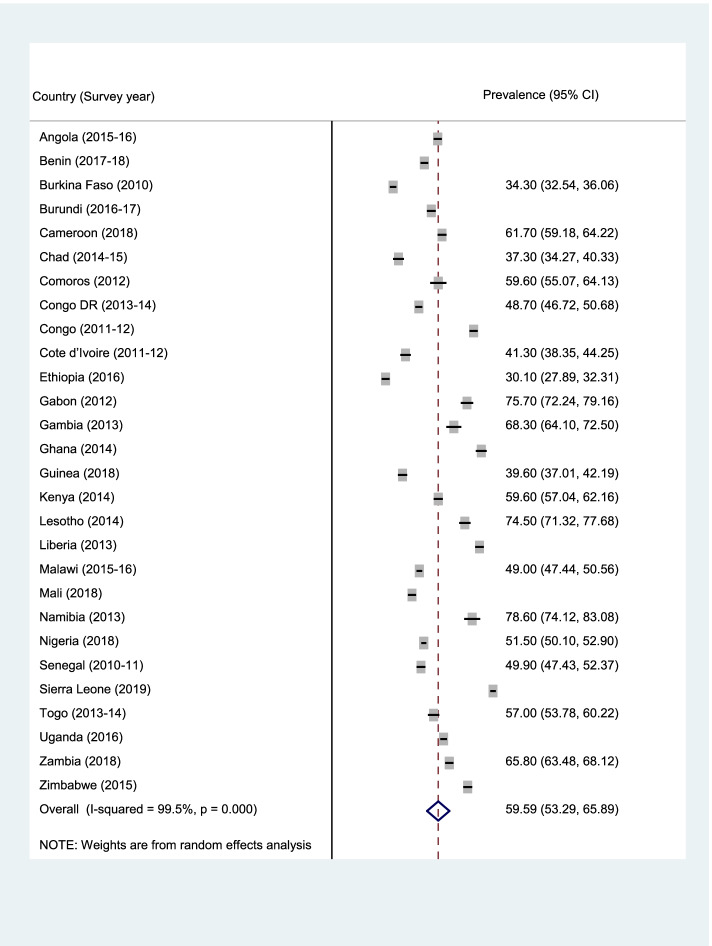
Prevalence of ANC among the young women in SSA

**Fig. 2:**
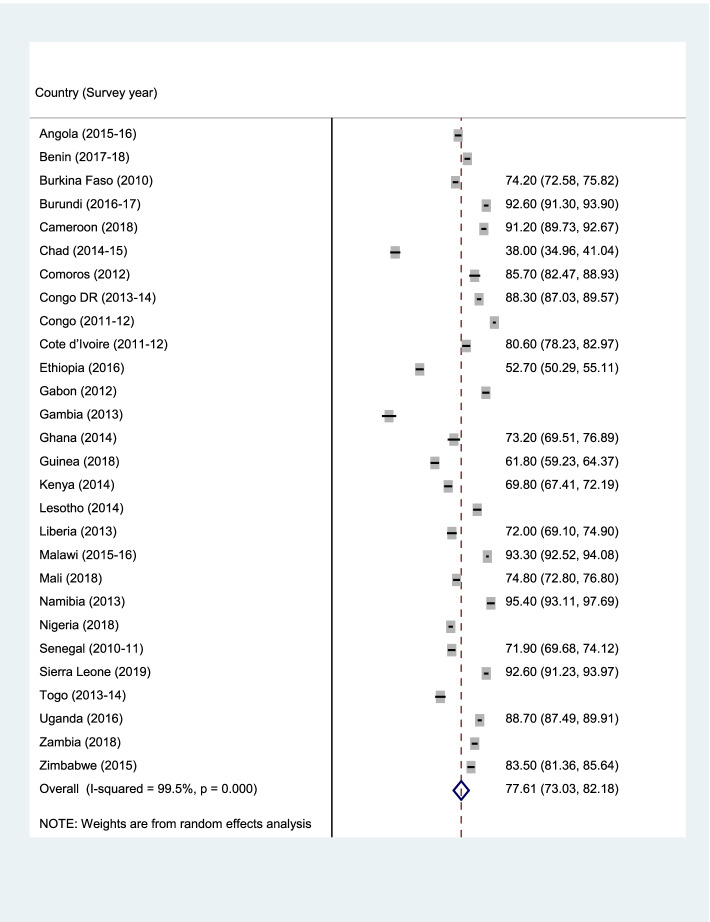
Prevalence of SBA among the young women in SSA

**Fig. 3:**
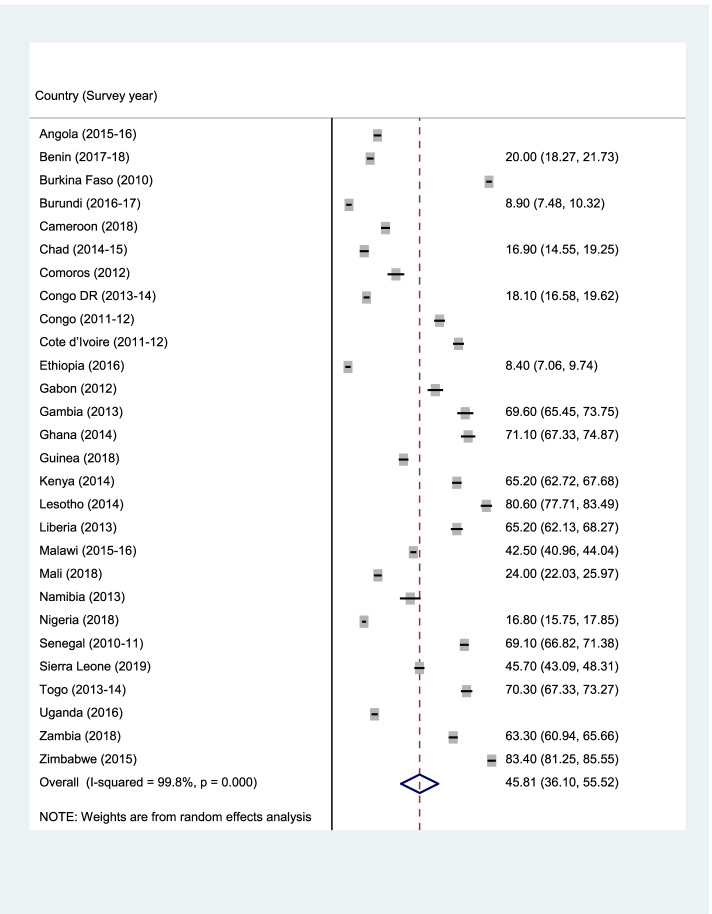
Prevalence of PNC among the young women in SSA

Data analyses were carried out using Stata version 16.0 (Stata Corporation, College Station, TX, USA). The analyses were performed at three levels. First, forest plot was used to summarise the prevalence of ANC, SBA, and PNC (Fig. 1–3). Next, the Pearson chi-square test was performed to examine the relationship between explanatory variables and ANC, SBA, and PNC (See Table 2). Finally, a multilevel binary logistic regrerssion was used to examine the determinants of ANC, SBA, and PNC (Table 2–4). Four models (Model I-IV) were built to examine the determinants. Model O showed the variance in ANC, SBA, and PNC attributed to the clustering of the primary sampling units (PSUs). Model I was fitted to contain the individual-level variables. Model II contained the household/community-level variables. Model III was finally fitted to contain all the individual and household/community level variables. We employed the Stata command “melogit” in fitting the four models. Akaike’s Information Criterion (AIC) tests was used to test for model comparison and fittness. The model with the least AIC was selected as the best fitted model. The results of the regression analyses were presented using adjusted odds ratio (aOR) with their respective 95% confidence intervals (CIs). Statistical significance was set at *p* < 0.05. A multicollinearity test was conducted using the Variance Inflation Factor (VIF) and we found that the minimum, maximum, and mean VIF were 1.00, 3.74, and 1.74 respectively. Hence, there was no evidence of high collinearity among the studied variables. The women’s sample weights (v005/1,000,000) were applied to obtain unbiased estimates, according to the DHS guidelines and the survey command 'SVY' in Stata was used to adjust for the complex sampling structure of the data in all the analyses.

#### Ethical considerations

Ethical permission was not sought for the present study since the DHS datasets used are publicly available. However, the DHS reports that ethical clearances were obtained from the Ethics Committee of ORC Macro Inc. as well as Ethics Boards of partner organisations of the various countries such as the Ministries of Health. The DHS follows the standards for ensuring the protection of respondents’ privacy. ICF International ensures that the survey complies with the U.S. Department of Health and Human Services’ regulations for the respect of human subjects. Further information about the DHS data usage and ethical standards are available at http://goo.gl/ny8T6X

## Results

### Prevalence of maternal healthcare utilisation

Figures 1, 2, and 3 (ANC, SBA, and PNC respectively) present the prevalence of maternal healthcare utilisation among young women in SSA. The highest prevalence of ANC, SBA, and PNC utilization was recorded in Sierra Leone (90.3%), Congo (97.5%), and Zimbabwe (88.4%) respectively. The lowest prevalence of ANC, SBA, and PNC utilisation were, however, respectively recorded in Ethiopia (30.1%), Gambia (32.3%), and Ethiopia (8.4%). Overall, the prevalence of maternal healthcare utilisation in SSA was 55.2%, 78.8%, and 40% for ANC, SBA, and PNC respectively.

Table 1 presents bivariable results on the predictors of maternal healthcare utilisation among young women in SSA. We found statistically significant relationships between maternal healthcare utilisation and maternal educational level, marital status, partner educational level, religion, parity, ownership of health insurance, mass media exposure, decision-making capacity, barrier to healthcare, wealth index, sex of household head, and residence. Maternal age was significantly related to ANC and PNC utilisation, partner’s age was significantly related to ANC and SBA utilization, and maternal current working status was only significantly related to SBA utilisation.

**Table 1:** Bivariable analysis of predictors of ANC, SBA, and PNC among young women in SSA

Variables	Weighted N	Weighted %	ANC		SBA		PNC	
			**Yes**	***P*-value**	**Yes**	***P*-value**	**Yes**	***P*-value**
**Maternal age**				< 0.001		0.133		< 0.001
15–19	9,348	21.3	51.8		78.1		37.0	
20–24	34,438	78.7	56.1		79.0		41.2	
**Maternal educational level**				< 0.001		< 0.001		< 0.001
No education	14,968	34.2	40.3		65.6		37.4	
Primary	15,385	35.1	55.3		81.3		38.2	
Secondary	12,787	29.2	71.3		90.2		46.1	
Higher	646	1.5	80.4		97.0		43.6	
**Marital status**				< 0.001		< 0.001		0.001
Married	33,391	76.3	52.6		76.8		41.0	
Cohabiting	10,395	23.7	63.6		85.0		37.8	
**Partner’s age**				< 0.001		< 0.001		0.070
15–24	8,685	19.8	55.7		83.3		39.0	
25–34	27,100	61.9	55.7		79.0		40.6	
35–44	6,142	14.0	54.1		74.0		39.8	
45 +	1,859	4.3	49.7		69.8		42.4	
**Partner educational level**				< 0.001		< 0.001		< 0.001
No education	13,644	31.2	40.6		66.0		39.1	
Primary	12,210	27.9	53.5		79.6		37.4	
Secondary	15,508	35.4	66.1		87.1		43.5	
Higher	2,424	5.5	76.4		93.2		41.3	
**Religion**				< 0.001		< 0.001		< 0.001
Christianity	25,032	57.2	60.7		84.8		41.2	
Islamic	16,936	38.7	48.3		71.0		38.4	
African Traditional	681	1.6	38.9		61.3		48.8	
No religion	951	2.2	47.0		68.7		46.8	
Others	185	0.4	56.4		84.8		24.5	
**Maternal current working status**				0.146		< 0.001		0.218
Not working	19,360	44.2	54.7		77.3		39.8	
Working	24,426	55.8	55.6		80.0		40.7	
**Parity**				< 0.001		< 0.001		< 0.001
1	20,460	46.7	59.6		83.9		42.2	
2	14,747	33.7	53.3		77.1		40.5	
3	6,317	14.4	48.8		71.0		37.0	
4 or more	2,262	5.2	45.9		65.2		30.9	
**Ownership of Health Insurance**				< 0.001		< 0.001		< 0.001
No	42,159	96.3	54.5		78.5		39.8	
Yes	1,627	3.7	73.4		85.7		52.1	
**Mass media exposure**				< 0.001		< 0.001		< 0.001
No	24,082	55.0	46.2		73.7		29.4	
Yes	19,704	45.0	60.6		81.9		46.9	
**Person who usually decides on respondent’s healthcare**				< 0.001		< 0.001		< 0.001
Respondent alone	5,648	12.9	61.5		81.7		47.9	
Respondent and partner	15,070	34.4	59.5		82.0		40.1	
Partner alone	22,491	51.4	50.8		75.9		38.3	
Someone else or other	577	1.3	52.8		78.6		48.4	
**Person who usually decides on large household purchases**				< 0.001		< 0.001		< 0.001
Respondent alone	4,598	10.5	61.1		81.5		43.1	
Respondent and partner	15,938	36.4	60.1		82.3		41.5	
Partner alone	22,418	51.2	50.6		75.8		38.3	
Someone else or other	832	1.9	54.5		77.5		55.5	
**Person who usually decides on visits to family or relatives**				< 0.001		< 0.001		< 0.001
Respondent alone	7,706	17.6	57.6		80.5		46.9	
Respondent and partner	17,952	41.0	58.6		80.8		40.5	
Partner alone	17,514	40.0	50.9		76.1		36.7	
Someone else or other	613	1.4	49.1		76.6		52.4	
**Barrier to healthcare**				< 0.001		< 0.001		< 0.001
No	15,473	35.3	62.4		83.6		43.1	
Yes	28,313	64.7	51.3		76.2		38.7	
**Wealth index**				< 0.001		< 0.001		< 0.001
Poorest	10,136	23.2	42.9		66.5		34.8	
Poorer	10,611	24.2	50.4		74.3		37.6	
Middle	9,169	20.9	56.1		79.8		40.3	
Richer	8,189	18.7	63.9		87.3		45.6	
Richest	5,680	13.0	72.1		95.2		47.2	
**Sex of household head**				< 0.001		< 0.001		< 0.001
Male	37,521	85.7	54.2		78.2		39.6	
Female	6,265	14.3	61.0		82.3		44.6	
**Residence**				< 0.001		< 0.001		< 0.001
Urban	12,725	29.1	70.0		90.6		46.8	
Rural	31,061	70.9	49.2		73.9		37.6	

^*^p-values obtained from chi-square test

### Determinants of maternal healthcare utilisation among young women in SSA

Tables 2, 3, and 4 present multivariable logistic regression analyses on the determinants of maternal healthcare utilization among young women in SSA for ANC, SBC, and PNC respectively. We found that young women in their early 20 s had higher odds of utilizing ANC (aOR = 1.19, 95% CI = 1.11–1.28), SBA (aOR = 1.15, 95% CI = 1.06–1.25) and PNC (aOR = 1.19, 95% CI = 1.11–1.28) than those in their late teens. Formal education was an important determinant of maternal healthcare utilization among young women in SSA. The probability of utilizing ANC and SBA among the young mothers increased with increasing level of maternal and partner’s education. Those with some formal education and whose partners also had some formal education were also all respectively more likely to utilise PNC than those with no formal education. Respondents who were unmarried also had lower odds of utilizing ANC, SBA, and PNC than those who were married.

**Table 2:** Mixed effect analysis of determinants of ANC among young women in sub-Saharan Africa

Variables	Model O	Model I aOR [95% CI]	Model II aOR [95% CI]	Model III aOR [95% CI]
**Fixed effects**				
**Year of survey**				
2010		1 [1.00, 1.00]		1 [1.00, 1.00]
2011		1.82^***^ [1.54, 2.17]		2.02^***^ [1.69, 2.40]
2012		1.90^***^ [1.60, 2.25]		2.96^***^ [2.42, 3.63]
2013		4.26^***^ [3.48, 5.20]		4.30^***^ [3.49, 5.29]
2014		1.31^***^ [1.13, 1.52]		2.01^***^ [1.69, 2.39]
2015		1.70^***^ [1.44, 2.01]		3.31^***^ [2.71, 4.14]
2016		1.08 [0.93, 1.24]		2.08^***^ [1.71, 2.52]
2017		1.51^***^ [1.25, 1.82]		2.87^***^ [2.27, 3.64]
2018		1.41^***^ [1.23, 1.61]		1.48^***^ [1.29, 1.70]
2019		3.15^***^ [2.68, 3.71]		5.13^***^ [4.29, 6.12]
**Maternal age**				
15–19		1 [1.00, 1.00]		1 [1.00, 1.00]
20–24		1.25^***^ [1.16, 1.33]		1.19^***^ [1.11, 1.28]
**Maternal educational level**				
No education		1 [1.00, 1.00]		1 [1.00, 1.00]
Primary		1.43^***^ [1.33, 1.55]		1.43^***^ [1.33, 1.55]
Secondary		2.01^***^ [1.84, 2.19]		1.81^***^ [1.66, 1.98]
Higher		2.31^***^ [1.69, 3.17]		1.90^***^ [1.38, 2.61]
**Marital status**				
Married		1 [1.00, 1.00]		1 [1.00, 1.00]
Cohabiting		1.16^***^ [1.07, 1.26]		1.13^**^ [1.05, 1.23]
**Maternal current working status**				
Not working		1 [1.00, 1.00]		1 [1.00, 1.00]
Working		1.14^***^ [1.08, 1.21]		1.16^***^ [1.10, 1.23]
**Partner’s age**				
15–24		1 [1.00, 1.00]		1 [1.00, 1.00]
25–34		1.06 [0.99,1.14]		1.03 [0.96,1.11]
35–44		1.15^**^ [1.05, 1.27]		1.09 [0.99, 1.20]
45 +		1.21^**^ [1.06, 1.39]		1.15^*^ [1.00, 1.31]
**Partner educational level**				
No education		1 [1.00, 1.00]		1 [1.00, 1.00]
Primary		1.34^***^ [1.24, 1.45]		1.35^***^ [1.25, 1.46]
Secondary		1.57^***^ [1.44, 1.71]		1.49^***^ [1.37, 1.63]
Higher		2.12^***^ [1.83, 2.46]		1.90^***^ [1.63, 2.22]
**Religion**				
Christianity		1 [1.00, 1.00]		1 [1.00, 1.00]
Islamic		0.96 [0.89, 1.04]		0.84^***^ [0.77, 0.92]
African Traditional		0.76^*^ [0.61, 0.95]		0.68^***^ [0.54, 0.85]
No religion		0.79^**^ [0.67, 0.95]		0.70^***^ [0.58, 0.84]
Others		0.68 [0.45,1.04]		0.69 [0.45, 1.06]
**Parity**				
1		1 [1.00, 1.00]		1 [1.00, 1.00]
2		0.76^***^ [0.71, 0.80]		0.78^***^ [0.73, 0.83]
3		0.68^***^ [0.63, 0.74]		0.72^***^ [0.67, 0.78]
4 or more		0.64^***^ [0.57, 0.71]		0.69^***^ [0.62, 0.78]
**Ownership of health insurance**				
No		1 [1.00, 1.00]		1 [1.00, 1.00]
Yes		1.56^***^ [1.33, 1.84]		1.47^***^ [1.25, 1.73]
**Mass media exposure**				
No		1 [1.00, 1.00]		1 [1.00, 1.00]
Yes		1.44^***^ [1.36, 1.52]		1.28^***^ [1.21, 1.36]
**Person who usually decides on respondent’s healthcare**				
Respondent alone		1 [1.00, 1.00]		1 [1.00, 1.00]
Respondent and partner		0.98 [0.89, 1.08]		0.97 [0.88, 1.08]
Partner alone		0.92 [0.84, 1.02]		0.92 [0.84, 1.02]
Someone else or other		0.85 [0.65, 1.10]		0.86 [0.66, 1.12]
**Person who usually decides on large household purchases**				
Respondent alone		1 [1.00, 1.00]		1 [1.00, 1.00]
Respondent and partner		1.06 [0.95, 1.19]		1.07 [0.96, 1.20]
Partner alone		1.04 [0.94, 1.16]		1.05 [0.95, 1.17]
Someone else or other		1.12 [0.91, 1.40]		1.08 [0.87, 1.35]
**Person who usually decides on visits to family or relatives**				
Respondent alone		1 [1.00, 1.00]		1 [1.00, 1.00]
Respondent and partner		0.97 [0.89, 1.06]		0.97 [0.89, 1.06]
Partner alone		0.89^*^ [0.81, 0.97]		0.90^*^ [0.82, 0.99]
Someone else or other		0.79^*^ [0.63, 0.99]		0.79^*^ [0.62, 0.99]
**Barrier to healthcare**				
No		1 [1.00, 1.00]		1 [1.00, 1.00]
Yes		0.77^***^ [0.72,0.81]		0.82^***^ [0.77, 0.87]
**Wealth index**				
Poorest			1 [1.00, 1.00]	1 [1.00, 1.00]
Poorer			1.32^***^ [1.23, 1.42]	1.20^***^ [1.12, 1.29]
Middle			1.52^***^ [1.40, 1.64]	1.27^***^ [1.17, 1.38]
Richer			1.81^***^ [1.66, 1.98]	1.42^***^ [1.30, 1.56]
Richest			2.28^***^ [2.03, 2.56]	1.64^***^ [1.45, 1.86]
**Sex of household head**				
Male			1 [1.00, 1.00]	1 [1.00, 1.00]
Female			1.23^***^ [1.14, 1.32]	1.10^*^ [1.02, 1.18]
**Residence**				
Urban			1 [1.00, 1.00]	1 [1.00, 1.00]
Rural			0.55^***^ [0.51, 0.60]	0.77^***^ [0.71,0.84]
**Subregions**				
Southern			1 [1.00, 1.00]	1 [1.00, 1.00]
Central			0.40^***^ [0.33, 0.49]	0.47^***^ [0.37, 0.59]
Eastern			0.42^***^ [0.35, 0.51]	0.48^***^ [0.39, 0.60]
Western			0.36^***^ [0.30, 0.43]	0.94 [0.75, 1.18]
**Random effect**				
PSU variance (95% CI)	0.136 [0.108 – 0.172]	0.086 [0.068 – 0.109]	0.105 [0.083 – 0.131]	0.088 [0.069 – 0.111]
ICC	0.039809	0.0254319	0.0308296	0.026065
Wald chi-square	Reference	2499.16***	918.37***	2878.27***
**Model fitness**				
Log-likelihood	-29,399.808	-26,819.808	-28,261.064	-26,509.159
AIC	58,803.62	53,723.62	56,544.13	53,120.32
N	43,786	43,786	43,786	43,786
Number of clusters	1,478	1,478	1,478	1,478

Exponentiated coefficients, 95% confidence intervals in brackets,* aOR* adjusted Odds Ratios, *CI* Confidence Interval, ^*^ *p* < 0.05, ^**^ *p* < 0.01, ^***^ *p* < 0.001; 1 = Reference category, *PSU* Primary Sampling Unit, *ICC* Intra-Class Correlation, *AIC* Akaike’s Information Criterion

**Table 3:** Mixed effect analysis of determinants of SBA among young women in sub-Saharan Africa

Variables	Model O	Model I aOR [95% CI]	Model II aOR [95% CI]	Model III aOR [95% CI]
**Fixed effects**				
**Year of survey**				
2010		1 [1.00, 1.00]		1 [1.00, 1.00]
2011		0.84 [0.64, 1.10]		1.04 [0.80, 1.36]
2012		1.48^**^ [1.16, 1.91]		1.46^**^ [1.11, 1.90]
2013		0.26^***^ [0.21, 0.34]		0.27^***^ [0.21, 0.36]
2014		0.40^***^ [0.32, 0.50]		0.38^***^ [0.30, 0.48]
2015		0.21^***^ [0.17, 0.27]		0.19^***^ [0.14, 0.25]
2016		0.65^***^ [0.52, 0.82]		0.59^***^ [0.45, 0.78]
2017		2.18^***^ [1.59, 3.00]		1.79^**^ [1.24, 2.58]
2018		0.66^***^ [0.55, 0.80]		0.77^**^ [0.64, 0.93]
2019		1.35^*^ [1.07, 1.71]		1.42^**^ [1.12, 1.81]
**Maternal age**				
15–19		1 [1.00, 1.00]		1 [1.00, 1.00]
20–24		1.21^***^ [1.11, 1.32]		1.15^**^ [1.06, 1.25]
**Maternal educational level**				
No education		1 [1.00, 1.00]		1 [1.00, 1.00]
Primary		1.63^***^ [1.49, 1.78]		1.50^***^ [1.37, 1.64]
Secondary		2.66^***^ [2.39, 2.97]		2.01^***^ [1.80, 2.25]
Higher		6.05^***^ [3.43, 10.66]		2.91^***^ [1.66, 5.10]
**Marital status**				
Married		1 [1.00, 1.00]		1 [1.00, 1.00]
Cohabiting		1.00 [0.89, 1.12]		0.98 [0.87, 1.09]
**Maternal current working status**				
Not working		1 [1.00, 1.00]		1 [1.00, 1.00]
Working		1.06 [0.99, 1.14]		1.17^***^ [1.09, 1.27]
**Partner’s age**				
15–24		1 [1.00, 1.00]		1 [1.00, 1.00]
25–34		0.90^*^ [0.82, 0.99]		0.85^**^ [0.78, 0.94]
35–44		0.90 [0.80, 1.02]		0.82^**^ [0.73, 0.93]
45 +		0.87 [0.74,1.03]		0.80^**^ [0.66, 0.92]
**Partner educational level**				
No education		1 [1.00, 1.00]		1 [1.00, 1.00]
Primary		1.33^***^ [1.21, 1.47]		1.25^***^ [1.12, 1.38]
Secondary		1.88^***^ [1.70, 2.10]		1.57^***^ [1.41, 1.75]
Higher		2.72^***^ [2.20, 3.37]		1.90^***^ [1.52, 2.37]
**Religion**				
Christianity		1 [1.00, 1.00]		1 [1.00, 1.00]
Islamic		0.67^***^ [0.60, 0.74]		0.62^***^ [0.56, 0.70]
African Traditional		0.46^***^ [0.36, 0.60]		0.53^***^ [0.42, 0.69]
No religion		0.57^***^ [0.46, 0.71]		0.63^***^ [0.50, 0.78]
Others		0.99 [0.62, 1.57]		0.89 [0.55, 1.45]
**Parity**				
1		1 [1.00, 1.00]		1 [1.00, 1.00]
2		0.65^***^ [0.60, 0.70]		0.66^***^ [0.61, 0.72]
3		0.51^***^ [0.46, 0.56]		0.55^***^ [0.49, 0.60]
4 or more		0.44^***^ [0.39, 0.51]		0.48^***^ [0.42, 0.55]
**Ownership of health insurance**				
No		1 [1.00, 1.00]		1 [1.00, 1.00]
Yes		0.86 [0.71, 1.05]		0.90 [0.73, 1.11]
**Mass media exposure**				
No		1 [1.00, 1.00]		1 [1.00, 1.00]
Yes		1.27^***^ [1.18, 1.37]		1.00 [0.92, 1.08]
**Person who usually decides on respondent’s healthcare**				
Respondent alone		1 [1.00, 1.00]		1 [1.00, 1.00]
Respondent and partner		1.05 [0.92, 1.19]		1.08 [0.94, 1.24]
Partner alone		0.99 [0.87, 1.12]		1.02 [0.90, 1.16]
Someone else or other		0.90 [0.65, 1.26]		0.94 [0.68, 1.30]
**Person who usually decides on large household purchases**				
Respondent alone		1 [1.00, 1.00]		1 [1.00, 1.00]
Respondent and partner		1.01 [0.87, 1.16]		1.01 [0.87, 1.18]
Partner alone		1.00 [0.88, 1.13]		1.03 [0.90, 1.18]
Someone else or other		0.95 [0.71, 1.27]		0.94 [0.70, 1.26]
**Person who usually decides on visits to family or relatives**				
Respondent alone		1 [1.00, 1.00]		1 [1.00, 1.00]
Respondent and partner		0.92 [0.82, 1.03]		0.94 [0.83, 1.05]
Partner alone		0.93 [0.84, 1.03]		0.95 [0.86, 1.06]
Someone else or other		0.93 [0.69, 1.26]		0.98 [0.73, 1.31]
**Barrier to healthcare**				
No		1 [1.00, 1.00]		1 [1.00, 1.00]
Yes		0.71^***^ [0.65, 0.77]		0.81^***^ [0.75, 0.88]
**Wealth index**				
Poorest			1 [1.00, 1.00]	1 [1.00, 1.00]
Poorer			1.46^***^ [1.35, 1.59]	1.33^***^ [1.22, 1.45]
Middle			1.86^***^ [1.69, 2.05]	1.56^***^ [1.41, 1.73]
Richer			2.72^***^ [2.41, 3.07]	2.23^***^ [1.96, 2.54]
Richest			6.51^***^ [5.32, 7.98]	4.88^***^ [3.93, 6.07]
**Sex of household head**				
Male			1 [1.00, 1.00]	1 [1.00, 1.00]
Female			1.15^**^ [1.05, 1.26]	1.15^**^ [1.05, 1.26]
**Residence**				
Urban			1 [1.00, 1.00]	1 [1.00, 1.00]
Rural			0.48^***^ [0.42, 0.55]	0.63^***^ [0.55, 0.72]
**Subregions**				
Southern			1 [1.00, 1.00]	1 [1.00, 1.00]
Central			0.47^***^ [0.37, 0.61]	0.47^***^ [0.35, 0.62]
Eastern			0.70^**^ [0.55, 0.88]	0.52^***^ [0.39, 0.70]
Western			0.32^***^ [0.26, 0.40]	0.38^***^ [0.29, 0.49]
**Random effect**				
PSU variance (95% CI)	0.367 [0.304 – 0.444]	0.375 [0.301 – 0.469]	0.369 [0.300 – 0.454]	0.366 [0.287 – 0.467]
ICC	0.100432	0.1024502	0.1008413	0.1001725
Wald chi-square	Reference	2509.24***	1123.47***	2750.49***
**Model fitness**				
Log-likelihood	-21,757.371	-19,073.326	-20,032.342	-18,484.939
AIC	43,518.74	38,230.65	40,086.68	37,071.88
N	43,786	43,786	43,786	43,786
Number of clusters	1,478	1,478	1,478	1,478

Exponentiated coefficients, 95% confidence intervals in brackets, *aOR* adjusted Odds Ratios, *CI* Confidence Interval, ^*^ *p* < 0.05, ^**^ *p* < 0.01, ^***^ *p* < 0.001, 1 = Reference category, *PSU* Primary Sampling Unit, *ICC* Intra-Class Correlation, *AIC* Akaike’s Information Criterion

**Table 4:** Mixed effect analysis of determinants of PNC among young women in sub-Saharan Africa

Variables	Model O	Model I aOR [95% CI]	Model II aOR [95% CI]	Model III aOR [95% CI]
**Fixed effects**				
**Year of survey**				
2010		1 [1.00, 1.00]		1 [1.00, 1.00]
2011		0.43^***^ [0.33, 0.57]		0.48^***^ [0.36, 0.63]
2012		0.23^***^ [0.18, 0.29]		0.43^***^ [0.34, 0.56]
2013		0.27^***^ [0.21, 0.35]		0.23^***^ [0.18, 0.30]
2014		0.13^***^ [0.11, 0.16]		0.23^***^ [0.19, 0.29]
2015		0.18^***^ [0.14, 0.23]		0.42^***^ [0.32, 0.54]
2016		0.05^***^ [0.04, 0.07]		0.09^***^ [0.07, 0.11]
2017		0.01^***^ [0.01, 0.02]		0.02^***^ [0.01, 0.02]
2018		0.05^***^ [0.04, 0.06]		0.05^***^ [0.04, 0.06]
2019		0.14^***^ [0.11, 0.17]		0.23^***^ [0.18, 0.29]
**Maternal age**				
15–19		1 [1.00, 1.00]		1 [1.00, 1.00]
20–24		1.28^***^ [1.18, 1.39]		1.16^***^ [1.07, 1.26]
**Maternal educational level**				
No education		1 [1.00, 1.00]		1 [1.00, 1.00]
Primary		1.28^***^ [1.17, 1.39]		1.21^***^ [1.11, 1.32]
Secondary		1.37^***^ [1.24, 1.51]		1.30^***^ [1.17, 1.44]
Higher		1.18 [0.91, 1.52]		1.07 [0.82, 1.39]
**Marital status**				
Married		1 [1.00, 1.00]		1 [1.00, 1.00]
Cohabiting		0.69^***^ [0.63, 0.75]		0.90^*^ [0.82, 0.99]
**Maternal current working status**				
Not working		1 [1.00, 1.00]		1 [1.00, 1.00]
Working		1.20^***^ [1.13, 1.28]		1.26^***^ [1.18, 1.34]
**Partner’s age**				
15–24		1 [1.00, 1.00]		1 [1.00, 1.00]
25–34		0.97 [0.91, 1.05]		0.98 [0.90, 1.06]
35–44		0.94 [0.85, 1.04]		0.96 [0.87, 1.06]
45 +		0.93 [0.80, 1.09]		0.94 [0.80, 1.10]
**Partner educational level**				
No education		1 [1.00, 1.00]		1 [1.00, 1.00]
Primary		1.11^*^ [1.02, 1.21]		1.08 [0.98, 1.18]
Secondary		1.08 [0.99, 1.19]		1.15^**^ [1.05, 1.27]
Higher		1.02 [0.88, 1.19]		1.06 [0.91, 1.24]
**Religion**				
Christianity		1 [1.00, 1.00]		1 [1.00, 1.00]
Islamic		0.88^**^ [0.81, 0.96]		0.76^***^ [0.69, 0.83]
African Traditional		0.98 [0.75, 1.29]		0.75^*^ [0.57, 0.99]
No religion		1.32^**^ [1.09, 1.61]		1.02 [0.83, 1.26]
Others		0.38^***^ [0.24, 0.61]		0.35^***^ [0.21, 0.59]
**Parity**				
1		1 [1.00, 1.00]		1 [1.00, 1.00]
2		0.89^***^ [0.84, 0.95]		0.94 [0.89, 1.01]
3		0.79^***^ [0.73, 0.86]		0.91^*^ [0.83, 0.99]
4 or more		0.62^***^ [0.54, 0.71]		0.76^***^ [0.66, 0.87]
**Ownership of health insurance**				
No		1 [1.00, 1.00]		1 [1.00, 1.00]
Yes		1.54^***^ [1.29, 1.85]		1.40^***^ [1.17, 1.69]
**Mass media exposure**				
No		1 [1.00, 1.00]		1 [1.00, 1.00]
Yes		1.51^***^ [1.42, 1.60]		1.40^***^ [1.32, 1.50]
**Person who usually decides on respondent’s healthcare**				
Respondent alone		1 [1.00, 1.00]		1 [1.00, 1.00]
Respondent and partner		0.76^***^ [0.69, 0.84]		0.87^**^ [0.78, 0.96]
Partner alone		0.70^***^ [0.64, 0.78]		0.86^**^ [0.77, 0.96]
Someone else or other		0.72^*^ [0.53, 0.98]		0.84 [0.61, 1.17]
**Person who usually decides on large household purchases**				
Respondent alone		1 [1.00, 1.00]		1 [1.00, 1.00]
Respondent and partner		1.14^*^ [1.02, 1.29]		1.06 [0.94, 1.20]
Partner alone		1.13^*^ [1.01, 1.27]		1.04 [0.92, 1.17]
Someone else or other		1.48^**^ [1.13, 1.95]		1.37^*^ [1.04, 1.82]
**Person who usually decides on visits to family or relatives**				
Respondent alone		1 [1.00, 1.00]		1 [1.00, 1.00]
Respondent and partner		1.09 [0.99, 1.19]		1.06 [0.96, 1.17]
Partner alone		0.83^***^ [0.76, 0.91]		0.82^***^ [0.74, 0.90]
Someone else or other		0.99 [0.76, 1.31]		0.88 [0.66, 1.17]
**Barrier to healthcare**				
No		1 [1.00, 1.00]		1 [1.00, 1.00]
Yes		0.78^***^ [0.73, 0.83]		0.86^***^ [0.81, 0.92]
**Wealth index**				
Poorest			1 [1.00, 1.00]	1 [1.00, 1.00]
Poorer			1.13^**^ [1.05, 1.22]	1.10^*^ [1.01, 1.19]
Middle			1.20^***^ [1.10, 1.30]	1.08 [0.98, 1.18]
Richer			1.33^***^ [1.21, 1.47]	1.14^*^ [1.02, 1.27]
Richest			1.32^***^ [1.18, 1.48]	1.09 [0.96, 1.25]
**Sex of household head**				
Male			1 [1.00, 1.00]	1 [1.00, 1.00]
Female			1.21^***^ [1.13, 1.30]	1.08 [1.00, 1.17]
**Residence**				
Urban			1 [1.00, 1.00]	1 [1.00, 1.00]
Rural			0.70^***^ [0.63, 0.76]	0.83^***^ [0.75, 0.91]
**Subregions**				
Southern			1 [1.00, 1.00]	1 [1.00, 1.00]
Central			0.18^***^ [0.15, 0.22]	0.20^***^ [0.16, 0.25]
Eastern			0.34^***^ [0.28, 0.41]	0.66^***^ [0.53, 0.82]
Western			0.41^***^ [0.34, 0.50]	0.93 [0.74, 1.17]
**Random effect**				
PSU variance (95% CI)	0.311 [0.253—0.383]	0.177 [0.140 – 0.225]	0.318 [0.257–0.394]	0.175 [0.139–0.220]
ICC	0.0864322	0.051193	0.088206	0.0504016
Wald chi-square	Reference	3198.44***	626.28***	3706.80***
**Model fitness**				
Log-likelihood	-28,519.644	-24,610.945	-27,772.427	-23,857.225
AIC	57,043.29	49,305.89	55,566.85	47,816.45
N	43,786	43,786	43,786	43,786
Number of clusters	1,478	1,478	1,478	1,478

Exponentiated coefficients, 95% confidence intervals in brackets, *aOR* adjusted Odds Ratios, *CI* Confidence Interval, ^*^ *p* < 0.05, ^**^ *p* < 0.01, ^***^ *p* < 0.001, 1 = Reference category, *PSU* Primary Sampling Unit, *ICC* Intra-Class Correlation, *AIC* Akaike’s Information Criterion

We found that multiparous young women had lower odds to utilise maternal healthcare than those who were primiparous. Young women who had health insurance were more likely to utilise ANC (aOR = 1.47, 95% CI = 1.25–1.73) and PNC (aOR = 1.40, 95% CI = 1.17–1.69) than those without health insurance. Respondents who were exposed to the mass media were also more likely to utilise ANC (aOR = 1.28, 95% CI = 1.21–1.36) and PNC (aOR = 1.40, 95% CI = 1.32–1.50) than those not exposed. Young women who indicated having a barrier to healthcare utilization, were actually less likely to utilise maternal healthcare (ANC: aOR = 0.82, 95% CI = 0.77–0.87; SBA: aOR = 0.81, 95% CI = 0.75–0.88; PNC: aOR = 0.86, 95% CI = 0.81–0.92). Young women who were in the richest wealth quintile were 1.64, 4.88, and 1.09 times respectively more likely to utilise ANC (95% CI = 1.45–1.86), SBA (95% CI = 3.93–6.07), and PNC (95% CI = 0.96–1.25) than young women in the poorest wealth quintile. In households where the head is a female, we found that women had higher odds of utilizing maternal healthcare than male-headed households. Rural dwellers were, however, less likely to utilise ANC and SBA than urban dwellers. The highest probabilities of ANC, SBA, and PNC utilization were respectively found in Sierra Leaone, Congo, and Zimbabwe.

## Discussion

Our study examined maternal healthcare utilisation and its determinants among young women in 28 SSA countries. We found that overall, the prevalence of maternal healthcare utilisation in SSA was 55.2%, 78.8%, and 40% for ANC, SBA, and PNC respectively. The findings revealed country-specific variations in the prevalence of maternal healthcare utilisation among young women with the highest prevalence and probabilities of ANC, SBA, and PNC being recorded in Sierra Leone, Congo, and Zimbabwe respectively.

The highest prevalence and probabilities of the respective components of maternal healthcare utilisation reported among young women in Sierra Leone, Congo, and Zimbabwe could be attributed to the various successful maternal health interventions that have been implemented in these countries to promote maternal healthcare among women overall, while reducing maternal and neonatal mortality ratios. For instance, Zimbabwe developed a National Maternal and Neonatal Health Road Map of 2007 which was launched in 2009. The intervention, which was highly successful, involved prioritising and scale up of evidence-based, up-to-date and cost-effective strategies and activities including the mobilisation of sufficient human, community health services, regular in-service training for health workers, and financial resources for maternal and neonatal healthcare [29]. Similarly, the Ministry of Public Health (MoPH) in Congo (DRC) implemented several maternal and child health interventions including the community-based maternal and child health project which focused on ensuring 4 + antenatal care among women ensuring successful skilled delivery through consistent health education and transportation systems for referrals [34, 35]. In 2010, the government of Sierra Leone through the Ministry of Health and Sanitation (MOHS) also implemented the Free Health Care Initiative (FHCI) which successfully provides comprehensive and free essential maternal health services, including antenatal, delivery, and PNC to women [36, 37].

Ideally, it is expected that women will attend ANC, give birth with skilled attendance, and then receive adequate PNC within 42 days after delivery [35, 38–40]. The high prevalence of SBA compared to the low ANC and PNC recorded in our study could be attributed to the recent concentration of national interventions in SSA on SBA at the expense of ANC and PNC [41–46]. For instance, the implementation of free delivery services, training and integration of Traditional Birth Attendants (TBA) into the orthodox maternal care, improved accessibility of facilities, establishment of community-based health planning and services (CHPS) to facilitate supervised and emergency skill delivery at the community level are interventions that have been vigorously implemented to improve SBA [41–46]. On the contrary, previous studies showing an almost universal ANC coverage in SSA [47–49] have probably led to reductions in the attention of health systems on this important component of maternal healthcare. Moreover, in SSA, especially in rural areas, mother in-laws as well as multigravida women have presumptions of knowing about the stages of pregnancy and overall ANC care, hence, they may neglect ANC attendance [43, 50–52].

The low prevalence of ANC and PNC utilisation recorded in our study is worrying as this militates against the achievement of SDG 3.1, and 3.8 targets of reducing the maternal mortality ratio to less than 70 maternal deaths per 100,000 live births, and achieving universal health coverage respectively in SSA [3, 48]. The very low maternal healthcare utilisation recorded in the majority of the countries we studied and especially in Ethiopia, Gambia, and Ethiopia could be ascribed to the myriad of institutional and contextual factors bedeviling the healthcare systems and the provision of maternal healthcare in the respective countries. For instance, lack of partner support, perceived unimportance of maternal healthcare, lack of trust in health facilities/perceived poor services provided, lack of accessibility and affordability of the services could explain the low prevalence of maternal healthcare utilisation recorded [53–56]. Others include negative attitude of some healthcare workers, lack of health decision making power by women, confidence in TBAs, perception of seeking maternal healthcare as unnecessary by mother-in-laws, and limited access to and utilisation of healthcare [52–59].

In this study, we found that young women who have some level of formal education were more likely to utilise maternal healthcare, especially ANC and PNC. This finding reveals the crucial role formal education in informing the health decisions of women. Similarly, we noted that women whose partners are formally educated had higher probabilities of utilising maternal healthcare. The observations made in this study corroborate previous studies which posited that maternal and partner’s formal education significantly increased the odds of women utilising prenatal care, hospital-based delivery, and PNC [60–62]. This observation could be attributed to the fact that pregnant women who are educated are more informed about the possible complications that can result from not seeking care before, during, and after delivery. Also, the finding where partner’s education increased maternal health service utilisation could be explained by the fact that educated partners know the importance of maternal health-seeking, and hence support their spouses to utilise maternal health service before, during, and after delivery. The perceived susceptibility to maternal complications and even death among educated mothers stimulates their willingness and confidence to seek professional care. This implies that efforts to improve maternal healthcare utilisation among women in SSA must adopt equal strategies that promote more health education on the benefits of seeking care or the consequences of not utilising maternal health among both women and partners especially among women and their partners who did not receive any formal education.

We found that multiparous women had lower odds to utilise maternal healthcare than those who were primiparous. This observation is in accordance with previous studies which found an inverse relationship between parity and healthcare utilisation among women [60, 63]. A study by Larsen, Exavery, Phillips, Tani, and Kanté [64], for instance, found that multiparous women were 84% less likely to utilise maternal health services than primiparous ones. This variation in the maternal healthcare utilisation between primiparous and multiparous women could be due to the fact that primiparous young women are at the highest risks of complications during pregnancy and delivery and for that matter end up utilising maternal healthcare more than multiparous women [65–67]. Besides, the observation of lower service utilisation among multiparous women could be due to the undesirable experiences including poor attitude of health professionals, long waiting time, high cost of service the multiparous might have experienced during their previous pregnancies or deliveries hence they probably become disgruntled with orthodox healthcare as found in previous studies [68–71].

Ownership of health insurance by young women increased the odds of PNC utilisation in our study. This finding is congruent to the findings from previous studies that health insurance improves healthcare utilisation as it provides financial risk protection to the women hence encourage them to seek postnatal orthodox health services [72–76]. This finding could be attributable to the financial protection offered by the possession of active health insurance, as previous studies have shown that out of pocket payments for maternal healthcare limit maternal health service utilisation among women [41, 77–79].

We also found that women who indicated having a barrier to healthcare utilisation could have lower odds of utilising maternal healthcare. Previous research has suggested that cultural and religious beliefs, family pressure, superstition, shyness, misconception, transportation, lack of family support, lack of autonomy, lack of access to maternal healthcare, poor quality of care, and religious beliefs serve as barriers to the utilisation of maternal healthcare services among women [80–84]. A study by Kea, Tulloch, Datiko, Theobald, and Kok [53] showed that traditions and beliefs including a tradition of hiding pregnancy at an early stage prevent women from seeking ANC. Also, the belief that a normal pregnancy with no seen complications needs no medical attention also prevents young women from seeking care [85, 86]. Out-of-pocket payments for maternal healthcare utilisation, especially among women without health insurance prevent many women in SSA from accessing maternal healthcare [87–89]. Furthermore, lack of transportation or financial power to afford transportation to the health facilities serves as a major barrier that hinders women’s service utilisation [90, 91].

In this study, we identified household wealth as an important determinant that could influence maternal healthcare utilisation among young women in SSA. We found in both our bivariable and multivariable analyses that the proportion and probability of utilising maternal healthcare increased with increasing wealth status among young women. This finding is parallel with the findings by previous studies [41, 65, 67, 92, 93]. A study by Yaya [77], for instance, demonstrated that maternal healthcare utilisation of ANC, SBA and PNC is positively and significantly linked to the wealth index of a household. The variation in maternal healthcare utilisation based on wealth status could be due to the fact that women from poor households are unable to afford the financial means to pay for transportation, for health cost at the facility, or even subscribe to health insurance, and hence are unable to utilise maternal healthcare [93–95].

We also found that in households where the head is a female, the odds of utilising maternal healthcare by young women were higher compared with male-headed households. This observation is congruent to postulations by previous studies [72, 96–98] and could be attributed to the fact that when women are at helm of the household, they have higher autonomy and decision-making power to make informed decision regarding the utilisation of maternal healthcare [96, 99, 100]. Also, women being heads of the household implies they have control over the household finances and have the purchasing power to utilise maternal services. Furthermore, it improves the support women receive or give to fellow women regarding maternal healthcare utilisation. This is because, like women themselves, they know and value the essence of effectively utilising maternal healthcare to protect their own lives and those of their babies [96, 101].

Place of residence was an important determinant of maternal healthcare utilisation in our study. Specifically, rural dwellers were less likely to utilise adequate ANC and SBA than urban dwellers. These findings are consistent with the findings of previous studies [29, 102–106] and could be explained by the fact that women residing in urban settings usually experience a multiplicity of health facilities [107–111] and, therefore, have easy access to healthcare due proximity of the health facilities, good nature of roads, and the availability of health professionals to attend to them in terms of ANC and SBA utilisation. Tanou and Kamiya [102] also posited that the non-availability of health facilities in rural areas and geographical inaccessibility of health facilities from rural settings are compounded by the poor nature of roads linking them to the facilities and thus constitute a major obstacle militating against maternal healthcare utilisation in SSA.

Our study had several limitations. First, as a cross-sectional study, we were unable to establish causal relationships between maternal healthcare utilisation and the explanatory variables. Also, because the responses by study participants were self-reported in the surveys, there is the possibility of social desirability bias on the part of the participants. Furthermore, the use of data from different time period could have affected the comparability of the results, however, these studies were the most recent of these countries.

Notwithstanding the limitations, a key strength of the study is that we used nationally representative data from the various SSA countries for our analyses. Also, our study is the first multi-country attempt using the nationally representative DHS data to simulatanuously examine the prevalence and determinants of the three main maternal health services (ANC, SBA and PNC) utilisation in SSA.

## Conclusion

While the utilisation of SBA was high, we found ANC and PNC utilisation among young women to be low in SSA. To accelerate progress towards attainment of the SDG targets related to reducing maternal mortality and achieving universal health coverage in SSA, our study recommends the adoption of interventions which have proven effective in some countries, by countries which recorded low maternal healthcare utilisation. The interventions include the implementation of free delivery services, improved accessibility of facilities, establishment of CHPS to facilitate supervised and emergency skill delivery at the community level, regular in-service training for health workers, and consistent public health education. These interventions could particularly focus on young women in the lowest wealth quintile, women who experience barriers to maternal healthcare utilisation, uneducated women, and young women from rural areas.

## Supplementary Information

**Additional file 1: Appendix 1.** Description of sample.

**Additional file 2.** STROBE Statement—checklist of items that should be included in reports of observational studies.

## Data Availability

The datasets generated and/or analysed during the current study are available in the DHS Program repository at https://dhsprogram.com/data/available-datasets.cfm
