# Supplementary material for: Prevalence and determinants of maternal healthcare utilisation among young women in sub-Saharan Africa: cross-sectional analyses of demographic and health survey data
Source: BMC Public Health. 2022 Apr 5;22:647. doi: 10.1186/s12889-022-13037-8 (PMC8981812; doi:10.1186/s12889-022-13037-8)
Supplement: Supplementary file 1 — Additional file 1: Appendix 1. Description of sample. [file 12889_2022_13037_MOESM1_ESM.pdf]

Appendix 1. Description of sample

Countries	Year of survey	Weighted N	Weighted %
Angola	2015-16	1,831	4.2
Benin	2017-18	2,052	4.7
Burkina Faso	2010	2,805	6.4
Burundi	2016-17	1,550	3.5
Cameroon	2018	1,428	3.3
Chad	2014-15	980	2.2
Comoros	2012	450	1.0
Congo DR	2013-14	2,451	5.6
Congo	2011-12	1,228	2.8
Cote D'Ivoire	2011-12	1,069	2.4
Ethiopia	2016	1,651	3.8
Gabon	2012	590	1.4
Gambia	2013	471	1.1
Ghana	2014	554	1.3
Guinea	2018	1,373	3.1
Kenya	2014	1,416	3.2
Lesotho	2014	721	1.7
Liberia	2013	922	2.1
Malawi	2015-16	3,961	9.1
Mali	2018	1,807	4.1
Namibia	2013	322	0.7
Nigeria	2018	4,900	11.2
Senegal	2010-11	1,578	3.6
Sierra Leone	2019	1,404	3.2
Togo	2013-14	907	2.1
Uganda	2014-15	2,614	6.0
Zambia	2018	1,600	3.7
Zimbabwe	2015	1,151	2.6
**All countries**		**43,786**	**100.0**
